# Stability of osteotomy in minimally invasive hallux valgus surgery with “8” shaped bandage during gait: a finite element analysis

**DOI:** 10.3389/fbioe.2024.1415617

**Published:** 2024-07-30

**Authors:** Xudong Sun, Ziyan Guo, Xuhan Cao, Binglang Xiong, Yaxin Pan, Weidong Sun, Zixing Bai

**Affiliations:** ^1^ Beijing University of Chinese Medicine, Beijing, China; ^2^ Wangjing Hospital, China Academy of Chinese Medical Sciences, Beijing, China; ^3^ Orthopedics and Traumatology Department of Shunyi Hospital, Beijing Traditional Chinese Medicine Hospital, Beijing, China

**Keywords:** finite element analysis, gait analysis, hallux valgus, minimally invasive, bandages, quasi-static

## Abstract

**Introduction:**

Hallux valgus, a common foot deformity, often necessitates surgical intervention. This study evaluates the biomechanical alterations in patients post-surgery, focusing on the efficacy of an “8” bandage fixation system to promote optimal recovery.

**Methods:**

A three-dimensional (3D) model was constructed using CT data from a patient with hallux valgus. A quasi-static finite element analysis (FEA) was conducted in conjunction with gait analysis to evaluate the biomechanical changes at the osteotomy site under “8” shaped bandage fixation following hallux valgus surgery. The effects of the “8” shaped bandage on the stability of the osteotomy site and bone healing were investigated at three load points during the gait cycle.

**Results:**

During the Loading Response (LR), Midstance (MSt), and Terminal stance TSt phases, the osteotomy end experienced maximum Von Mises stresses of 0.118, 1.349, and 1.485 MPa, respectively. Correspondingly, the maximum principal stresses, all of which were compressive along the *Z*-axis, were 0.11662 N, 1.39266 N, and 1.46762 N, respectively. Additionally, these phases showed a maximum relative total displacement of 0.848 mm and a maximum relative shear displacement of 0.872 mm.

**Conclusion:**

During the stance phase, the osteotomy end of the first metatarsal is predominantly subjected to compressive stress, with the relative displacement within the safe range to promote healing. The application of an “8” bandage for external fixation after surgery can maintain the dynamic stability of osteotomy sites post-minimally invasive hallux valgus correction during the gait cycle, thereby promoting the healing of the osteotomy ends.

## 1 Introduction

Hallux valgus is a common condition in foot and ankle surgery, characterized primarily by the lateral deviation of the great toe, and often accompanied by erythema, swelling, pain at the bunion site, and restricted range of motion. Recent epidemiological meta-analyses (Cai et al., 2023) indicate that the global prevalence of hallux valgus is approximately 19%. Conservative treatment options for hallux valgus are generally limited in effectiveness, often necessitating surgical correction. The treatment method used in this study combines minimally invasive surgery, traditional Chinese manual correction techniques, and the “8” shaped bandage external fixation. This approach has already demonstrated significant clinical effectiveness, showing marked improvements in radiographic parameters, clinical symptoms, and reoperation rates (Wen et al., 2018). While many current studies (Baig et al., 2017; Klauser, 2019; Lucattelli et al., 2020; Füssenich er al., 2023) on hallux valgus surgery employ screws, plates, and K-wires for fixation, our method uses bandage external fixation, which has been shown to have comparable clinical efficacy. Additionally, this method does not require metal implants, resulting in fewer complications, such as skin and soft tissue irritation, and eliminating the need for subsequent hardware removal surgeries. It also allows for immediate weight-bearing and walking, and is more cost-effective (Ji et al., 2022).

While the clinical efficacy of “8” bandage fixation is acknowledged (Sun et al., 2010), the stability of the osteotomy site remains a topic of debate. A cadaver study demonstrated that the stability provided by the “8” bandage is comparable to K-wire fixation (Tai et al., 2023). Static FEA also revealed that using an “8” bandage for external fixation can reduce displacement at the osteotomy site (Xie et al., 2020). However, Xie et al. (2023) and Guo et al. (2022) and their team reported from static FEA that external bandage fixation may offer less effective stabilization compared to screw, steel plate, K-wire internal fixation.

**FIGURE 1:**
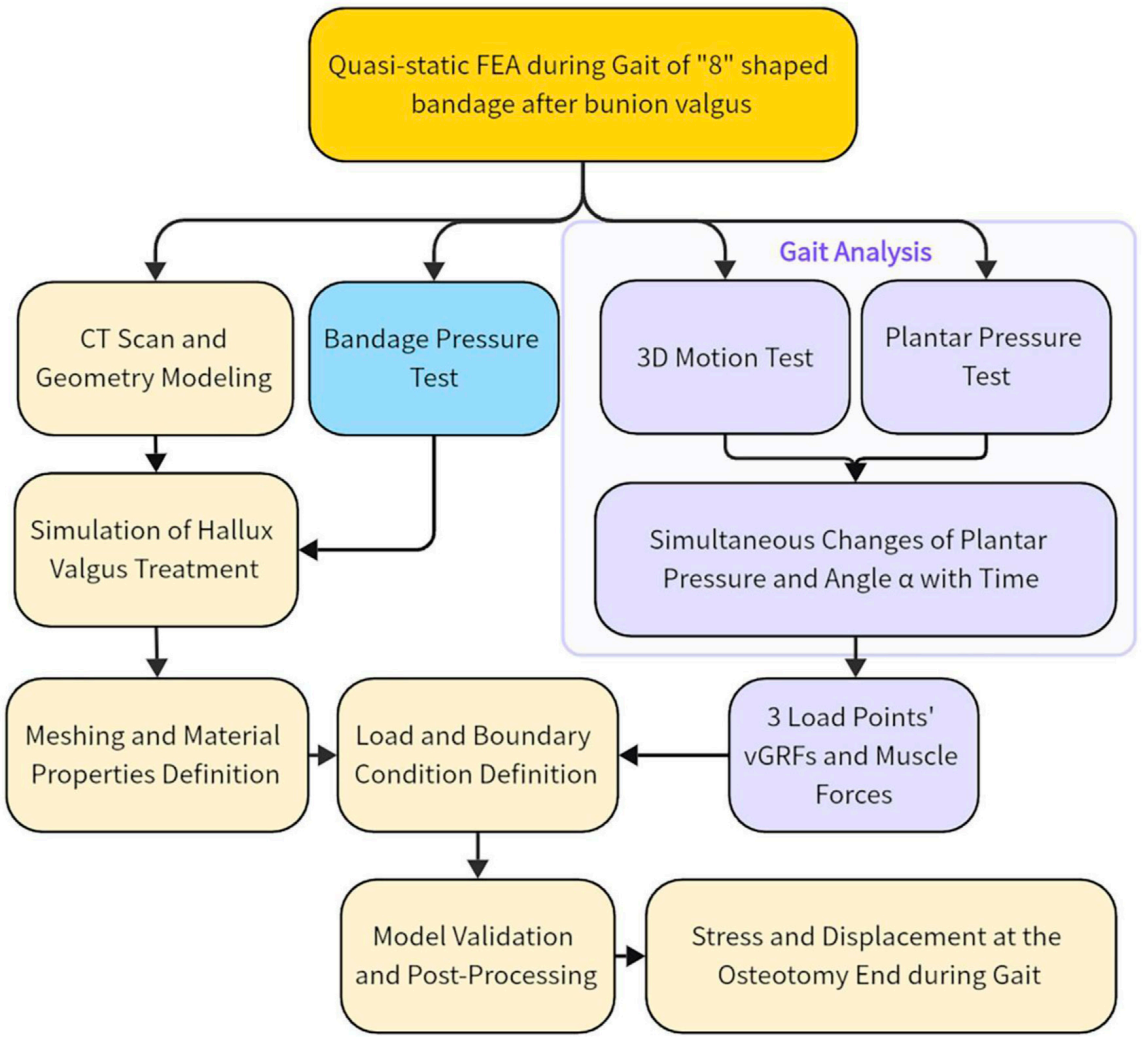
Technology roadmap. Abbreviation Explanations: 3D, Three-dimensional; FEA, Finite element analysis; vGRF, Vertical ground reaction force.

However, these studies were all conducted under static conditions and did not consider the postoperative weight-bearing walking situation. FEA allows for a detailed examination of the mechanical behavior of the foot under dynamic conditions, providing insights into stress distribution and displacement. In gait analysis, the timing of peak plantar pressure can be used as a marker to simplify the process of dynamic FEA. So this study employs a quasi-static FEA by selecting specific key points during the gait cycle, which can capture critical biomechanical changes while simplifying the computational complexity of dynamic finite element analysis. The technical flowchart outlining the steps of our methodology is presented in Figure 1.

Utilizing gait analysis and FEA, this study aimed to investigate the osteotomy site stability of “8” shaped bandage fixation during gait following minimally invasive treatment of hallux valgus, integrating traditional Chinese and Western medicine.

## 2 Methods

### 2.1 Participants

A 35-year-old female volunteer (167 cm in height, 50 kg in weight) underwent weight-bearing X-ray and CT examinations of her right foot. The results showed a hallux valgus angle of 20°, an intermetatarsal angle of 11°, a proximal articular set angle of 4°, and a distal articular set angle of 5°. A senior foot and ankle surgeon diagnosed her with hallux valgus and confirmed the absence of other foot deformities, and any history of foot surgery or trauma. The volunteer walked barefoot without discomfort after the bandage, put on during surgical treatment, was removed 6 weeks post-operation. There was no abnormal movement at the osteotomy site, and X-rays showed satisfactory alignment and congruence after correction, with callus formation present. She underwent gait and 3D motion testing. The study adhered to the ethical standards of the Ethics Committee of Wangjing Hospital, China Academy of Chinese Medical Sciences, with approval reference number 2013-03-21. Written informed consent was obtained from her after explaining the study procedures and potential risks.

### 2.2 Treatment

**FIGURE 2:**
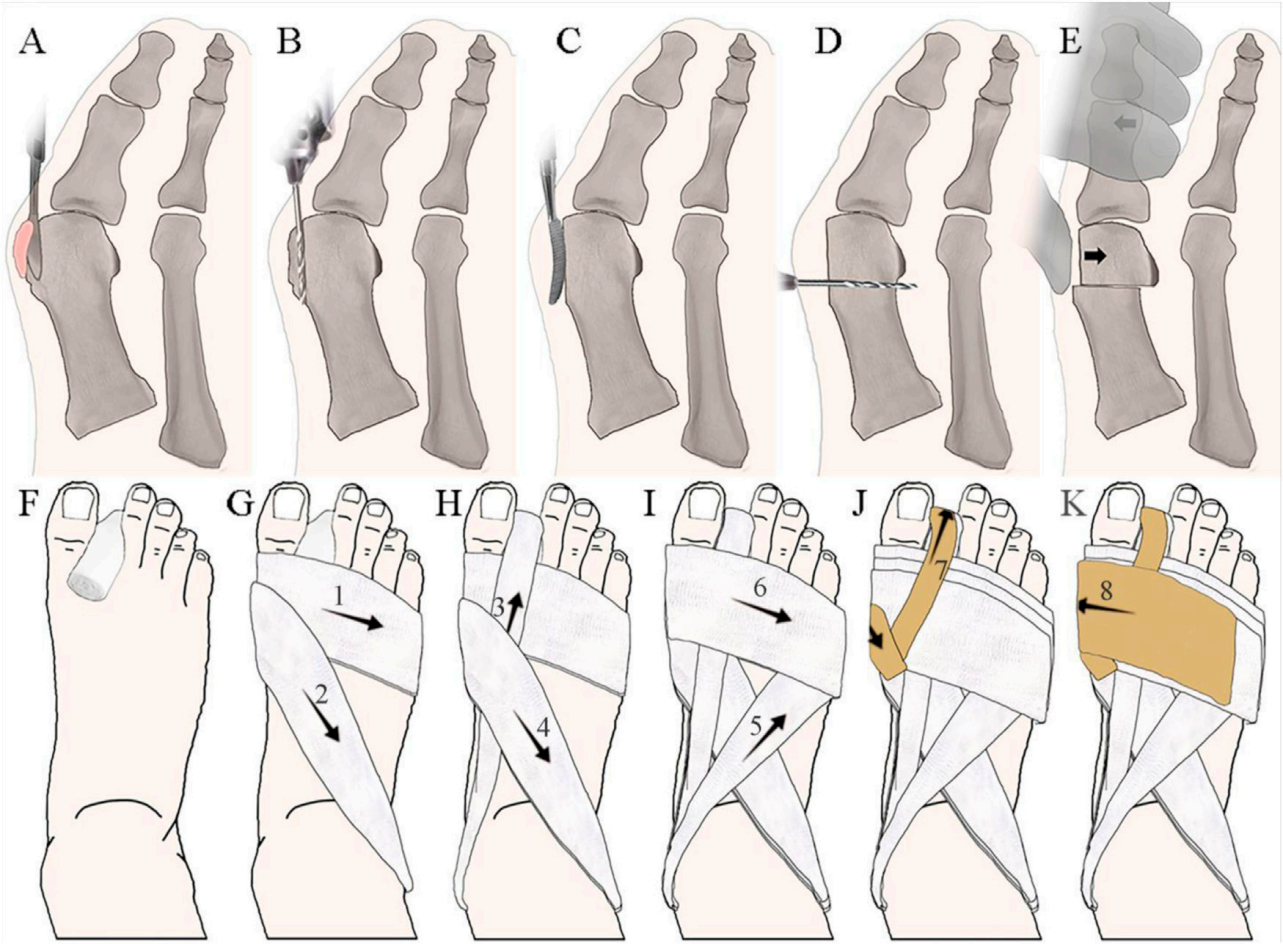
Treatment Integrated of Chinese Traditional and Western Medicine for Hallux Valgus. **(A)** Bursa excision; **(B)** Medial eminence resection; **(C)** Bone rasping; **(D)** Metatarsal osteotomy; **(E)** Manual correction; **(F–K)** “8” shaped bandage fixation method, where numbers represent the order of wrapping.

Minimally invasive techniques integrated of Chinese and Western medicine for hallux valgus primarily consists of six steps (Figure 2): bursa excision, medial eminence resection, bone rasping, metatarsal osteotomy, manual correction, and external fixation with “8” shaped bandage. The metatarsal osteotomy is performed at the head-neck junction of the first metatarsal. In the horizontal plane, the osteotomy plane forms a 70° angle with the axis of the first metatarsal, and a 60° angle in the sagittal plane, extending from distal medial-dorsal to proximal lateral-plantar. After the manual correction of the hallux, an appropriately sized gauze rolls separates the great and second toes. A “8” shaped bandage stabilizes the hallux in a 5° varus and slightly downward position, followed by adhesive tape reinforcement.

### 2.3 Instruments and equipment

This finite element study utilized the SOMATOM Definition Edge 64-slice spiral CT scanner by Siemens AG, Germany; the real-time pressure testing and analysis system, Tactilus 4.0 from Sensor Products Inc., American; the 3D visualization database, VTK 6.0 from Kitware Inc., United States ; the 3D digital reverse engineering software, Geomagic Studio 12.0, by Raindrop Geomagic, United States ; the software CATIA V5 from Dassault Systèmes, France; and the FEA software, ANSYS 14.0, from ANSYS, Inc., United States . Foot pressure measurement was performed using the Footscan 1-m platform pressure plate, produced by RSscan, Belgium. The 3D motion measurement system employed was the NDI Optotrak Certus (Northern Digital Inc., Canada). Tests were conducted at the Department of Orthopedics and Traumatology II, Wangjing Hospital of the China Academy of Chinese Medical Sciences, and at the Key Laboratory of Prosthetics & Orthotics Technology, Ministry of Civil Affairs, Beijing Vocational College of Social Management.

### 2.4 Gait dynamics index collection

Gait analysis was divided into two parts: plantar pressure test and 3D movement test. To collect gait data, we set up two cameras 5 m apart, using the NDI Optotrak Certus 3D motion measurement system. We placed ten markers on key anatomical landmarks: the medial side of the big toe, the medial side of the first metatarsal head, the lateral side of the fifth metatarsal head and base, the medial and lateral sides of the calcaneus, the tips of the medial and lateral malleoli, the posterior aspect of the mid-shaft of the tibia on the medial side, and the lateral aspect of the mid-shaft of the fibula.

**FIGURE 3:**
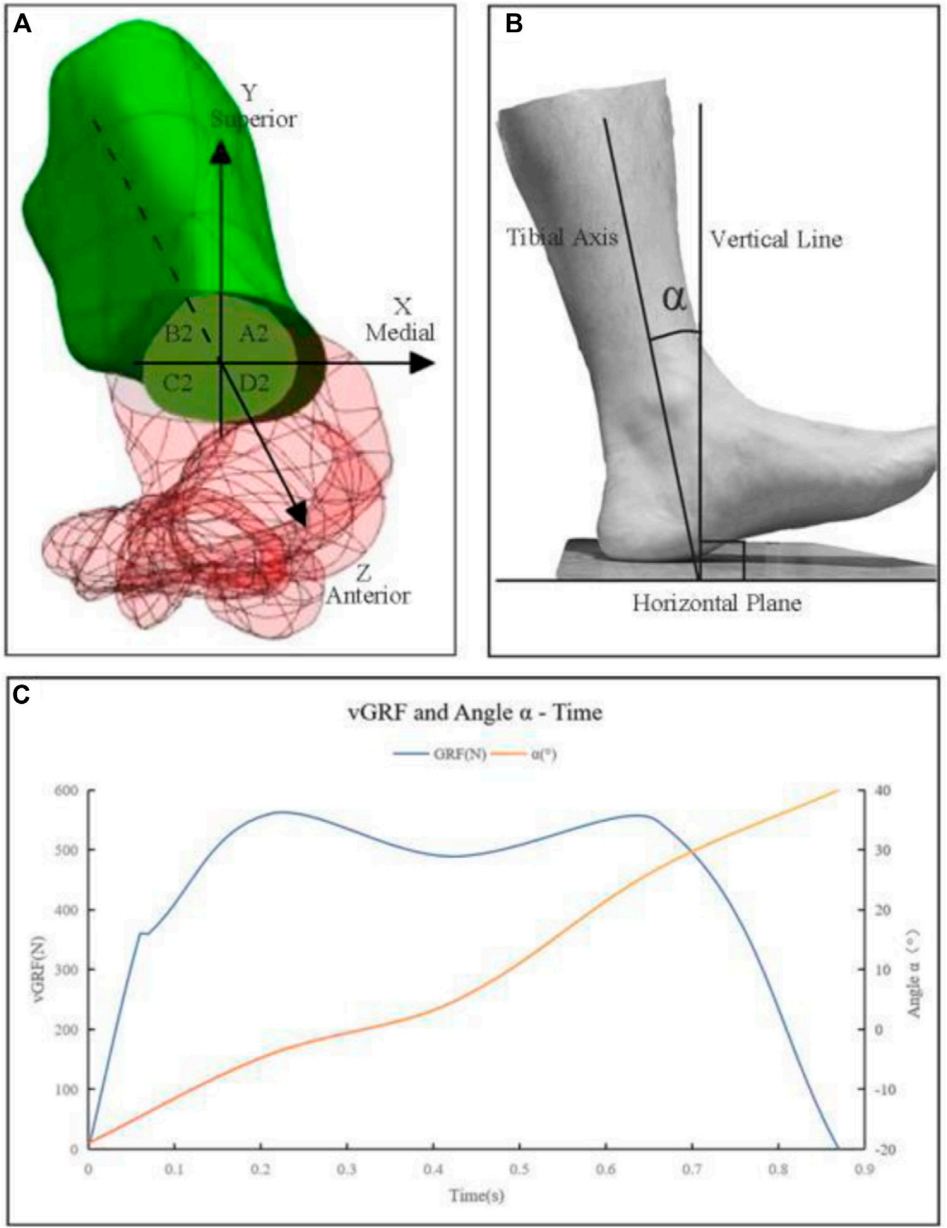
**(A)** The first metatarsal bone positioned within the established spatial coordinate system. A2, B2, C2, and D2 are nodes on the proximal osteotomy surface, corresponding to the medial superior, lateral superior, lateral inferior, and medial inferior regions of the proximal osteotomy surface. Correspondingly, there are four nodes on the distal osteotomy surface, denoted as A1, B1, C1, and D1. **(B)** α—Sagittal plane angle between the tibial shaft and the vertical line; **(C)** Graph of vGRF and α - Time.

Volunteer stood barefoot, arms down and slightly apart, feet shoulder-width apart. They walked a set path three times at a comfortable pace to ensure all markers were visible. Plantar pressure test and 3D motion test were performed simultaneously to obtain the change of plantar pressure and its relationship with limb Angle (Figure 3C). Since the ground reaction force in this study was primarily vertical, the horizontal components were ignored to simplify the analysis, focusing only on the Vertical ground reaction force (vGRF).

Muscle forces, specifically those of the extrinsic foot muscles, were derived from the α angle based on literature review and subsequently simulated as mechanical loads (Arnold et al., 2000; Hida et al., 2017; Wong et al., 2018; Fröberg et al., 2020).

### 2.5 Selection of load points

Drawing from the research by Perry and Burnfield (2010) and Yu et al. (2010), the complete gait cycle encompasses phases such as Initial Contact (IC), Loading Response (LR), Mid-Stance (MSt), Terminal Stance (TSt), Pre-Swing (PSw), Initial Swing (ISw), Mid-Swing (MSw), and Terminal Swing (TSw). These studies highlighted that vGRF during foot-ground contact show two distinct peaks separated by a valley, termed the LR peak, MSt valley, and TSt peak. The LR peak corresponds to initial contact and weight acceptance, the MSt valley marks the body’s passage over a stationary foot, and the TSt peak signifies the forceful push-off for forward motion. These phases are pivotal in understanding how the body’s biomechanics adapt for stability and efficiency during walking.

This study aimed to assess the stability of the osteotomy site under an “8” shaped bandage fixation following hallux valgus surgery throughout the gait cycle. Given that biomechanical changes at the osteotomy site are closely linked to the vGRF of the entire foot, the two peaks and one valley identified in gait analysis were deemed critical load points. These points were utilized in FEA to explore the biomechanical changes at the osteotomy site throughout the gait cycle.

### 2.6 “8” shaped bandage external fixation pressure test

Pressure values between location of bandage fixation and metatarsophalangeal joints in balanced standing were obtained by Tactilus 4.0. The device displays the value of force loading on the sensor with a diameter of 4 mm. The skin around the osteotomy site was divided into five measurement regions: the plantar, dorsal, and medial sides of the 1st metatarsal head; the central part and lateral side of the toe web. The pressure values were averaged over three walking trials and applied as loading condition in FE model.

### 2.7 Establishment of the quasi-static finite element model

#### 2.7.1 Imaging data collection

Perform a CT scan of the volunteer’s right foot with a slice thickness of 0.625 mm and no gaps to obtain CT image data of the foot, which should be exported in DICOM format. During the scan, the subject should be in a supine position with the ankle joint in a neutral position.

#### 2.7.2 Geometry modeling

Using VTK 6.0, DICOM files were read and chromatic values were set. Through a combination of automatic image segmentation and manual adjustments, a 3D rendered model of the foot’s skeletal structure was obtained. The skeletal, soft tissue and skin point cloud files were imported into Geomagic Studio 12.0 for surface reconstruction to optimize the model characteristics. Boolean operations facilitated the creation of cartilage and joint connections. By integrating anatomical data, the muscle was added to the model, resulting in a preliminary finite element model of hallux valgus.

#### 2.7.3 Treatment simulation on the model

The treatment process for hallux valgus was simulated on the constructed finite element model using CATIA V5 software. The simulation encompassed medial eminence resection, metatarsal osteotomy, and manual correction (Bai et al., 2020). The specifics of the first two procedures were as previously described. For the manual correction, the distal end of the metatarsal osteotomy was translated laterally by 3 mm, and the hallux was aligned in a 5-degree varus position.

#### 2.7.4 Meshing and material properties definition

The 3D model was imported into ANSYS 14.0 for meshing. An element size of approximately 1.5 mm was used in areas requiring higher accuracy (joints, regions around osteotomies), while a coarser mesh with an element size of approximately 3 mm was applied to less critical regions. Ligaments and the plantar fascia were defined as tension-only spring elements. Micromotion joints were modeled using elastic cartilage fusion, and metatarsophalangeal and interphalangeal joints were connected using nonlinear hyperelastic cartilage. Cortical bone, cancellous bone, marrow cavity, and fat pad were modeled as isotropic elastic materials. The properties of bone tissue, external soft tissue capsule, articular cartilage, and ligaments were obtained from literature. Detailed definitions are provided in Table 1 (Huiskes, 1982; Ka et al., 1998; Gefen et al., 2000; Tao et al., 2008; Bian et al., 2015; Morales-Orcajo et al., 2017).

**TABLE 1:** Key finite element model material properties.

Tissue	Element type	Young’s modulus E (MPa)	Poisson’s ratio
Cortical Bone	3D-tetrahedra	7,300	0.3
Cancellous Bone	3D-tetrahedra	100	0.3
Cartilage	3D-tetrahedra	1	0.4
Ligament	Tension-only truss	260	0.3
Fascia	Tension-only truss	350	0.49
Ground	3D-tetrahedra	100,000	0.3

#### 2.7.5 Load and boundary condition definition

Let α be the angle between the tibia and the vertical line, as depicted in Figure 3B. Using gait analysis, three load points were defined based on the vGRF and the corresponding α. From these Angles α, muscle forces were derived and then simulated as mechanical loads (Arnold et al., 2000; Hida et al., 2017; Wong et al., 2018; Fröberg et al., 2020). At the LR point, with an α of −4.2s, the vertical force was 280 N and the muscle loads were 88 N (Achilles tendon), 61.2 N (tibialis anterior muscle), and 151 N (tibialis posterior muscle). At the MSt point, with an α of 4.4°, the vertical force was 244.5 N and the muscle loads were 930 N (Achilles tendon), 83.5 N (tibialis posterior muscle), 58.3 N (peroneus longus muscle), 55 N (peroneus brevis muscle), and 48 N (flexor digitorum longus muscle). At the TSt Point, with an α of 26.8°, the vertical force was 274 N and the muscle loads were 1532 N (Achilles tendon), 236.1 N (flexor hallucis longus muscle), 82.2 N (flexor digitorum longus muscle), and 83.2 N (peroneus brevis muscle). The forces from other intrinsic and extrinsic muscles were neglected.

Based on the pressure test results, mechanical loading was applied to various regions to simulate the effect of bandage fixation. The averaged pressure values for each loading region are as follows: Plantar side of the head of the first metatarsal: 0.0638 MPa; Dorsal side of the head of the first metatarsal: 0.0558 MPa; Medial side of the head of the first metatarsal: 0.032 MPa; Center of the toe web: 0.072 MPa; Lateral side of the toe web: 0.0462 MPa.

According to the principle of equivalent exchange between loads and vGRFs, a concentrated upward force was applied at the center of pressure, passing through the support structure surface. The ankle joint surface was fully constrained, maintaining a neutral position during balanced standing. Granulation tissue, set to 3 mm thick, was used to fill the osteotomy gap, and the friction coefficient at the osteotomy site was set at 0.66 (Bai et al., 2020), as provided by prior project research. The friction coefficient between the sole and the ground is 0.6 (K et al., 2014).

#### 2.7.6 Establishment and verification of the finite element model

By comparing the plantar stress of the hallux valgus foot finite element model with the plantar pressure measured by the Footscan force plate, it was found that the pressure concentration areas shown by both methods are generally consistent (mainly, especially the values under the first metatarsal head are quite close (1.008 Vs. 0.977 MPa). This indicates that the model is fundamentally reliable and effective for simulating weight-bearing walking conditions with an “8” shaped bandage after hallux valgus surgery (Bai et al., 2020).

A 3D finite element model of a hallux valgus foot was established. This finite element model includes 28 bones, 56 ligaments, cartilage, skin, plantar fascia, and other soft tissues. The soft tissue mesh consists of 160,817 elements and 34,859 nodes, while the bone mesh consists of 289,124 elements and 58,313 nodes. Simulations were conducted for three load points.

### 2.8 Main observation indicators

A spatial coordinate system was established at the center point of the first metatarsal osteotomy surface. The *Z*-axis was parallel to the axis of the proximal part of the first metatarsal, pointing anteriorly; the *X*-axis was perpendicular to the *Z*-axis in the horizontal plane, pointing medially; and the *Y*-axis was perpendicular to the XZ plane, pointing superiorly. The selected nodes on the distal osteotomy surface were labeled A1 (medial superior), B1 (lateral superior), C1 (lateral inferior), and D1 (medial inferior), with corresponding nodes on the proximal surface labeled A2, B2, C2, and D2. Displacement was considered positive when it aligned with the axis direction and negative when it was in the opposite direction (Figure 3A).

Using ANSYS 14.0 software for solving and post-processing. Output the von Mises stress and total displacement at the osteotomy site, as well as the principal stresses and displacements along the XYZ axes, and visualize with corresponding contour plots.

## 3 Result

### 3.1 Gait analysis

The results of the gait analysis, which include plantar pressure testing and 3D motion testing, indicate that the volunteer’s stance phase lasted 0.87 s, during which two peaks and one valley in plantar pressure were observed. The LR peak was 560 N, occurring at 0.21 s in the gait cycle, with an α of −0.42°; the MSt valley was 489 N, occurring at 0.42 s in the gait cycle, with an α of 4.4°; the TSt peak was 548 N, occurring at 0.66 s in the gait cycle, with an α of 26.8°.

### 3.2 Stress outcomes from FEA

**FIGURE 4:**
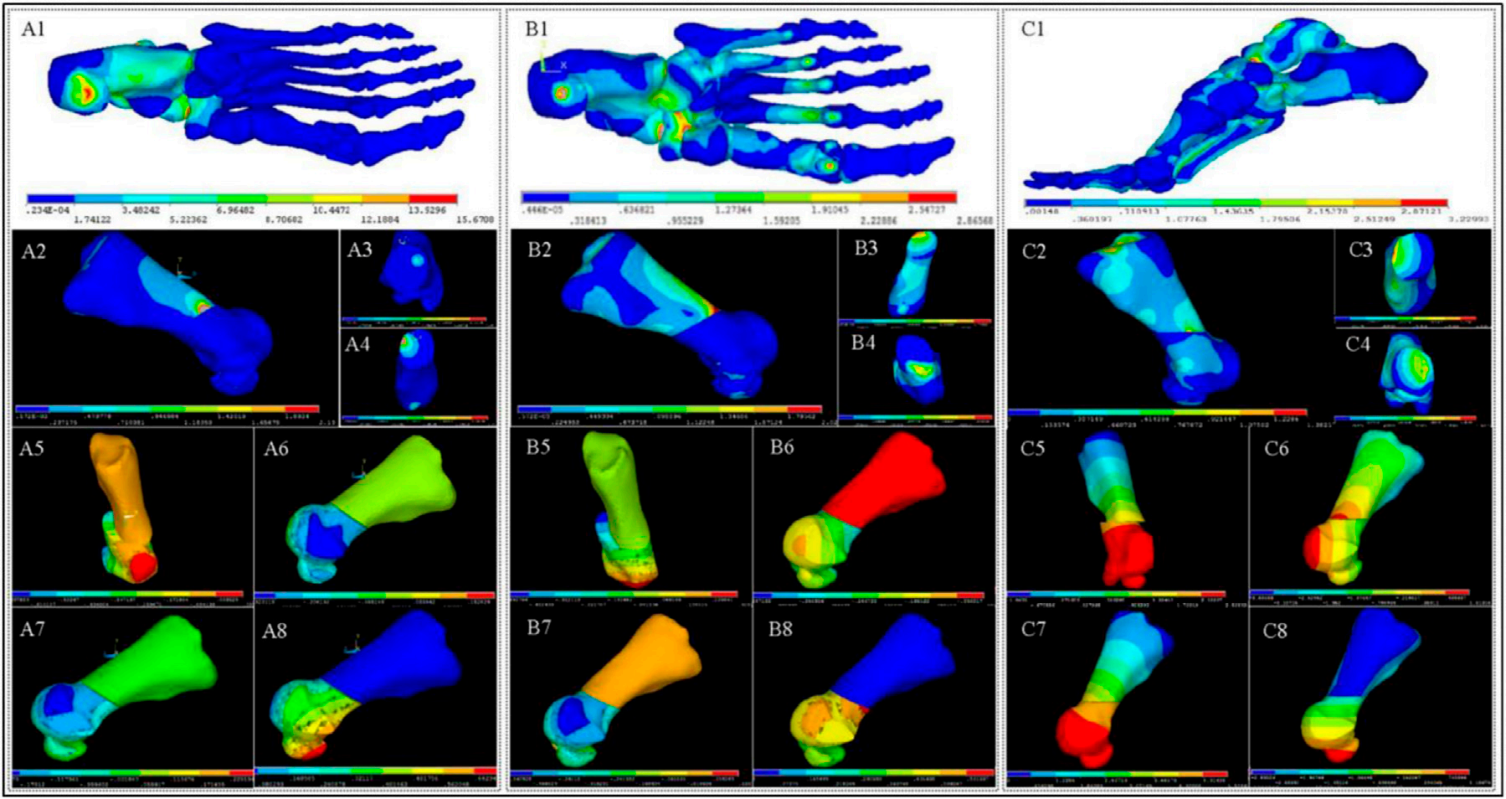
Stress and Displacement Contour Maps in Foot and First Metatarsal Bone. **(A1–A4)** depict stress contours in the foot **(A1)** and 1st metatarsal bone **(A2)**, highlighting proximal **(A3)** and distal **(A4)** osteotomy regions during the LR phase. **(A5–A8)** show displacement contours of the 1st metatarsal bone along the *X*-axis **(A5)**, *Y*-axis **(A6)**, *Z*-axis **(A7)**, and overall displacement **(A8)**. **(B1–B8)**, **(C1–C8)** present stress and displacement contours for the MSt **(B1–B8)**, Pr **(C1–C8)** phases, respectively.

**FIGURE 5:**
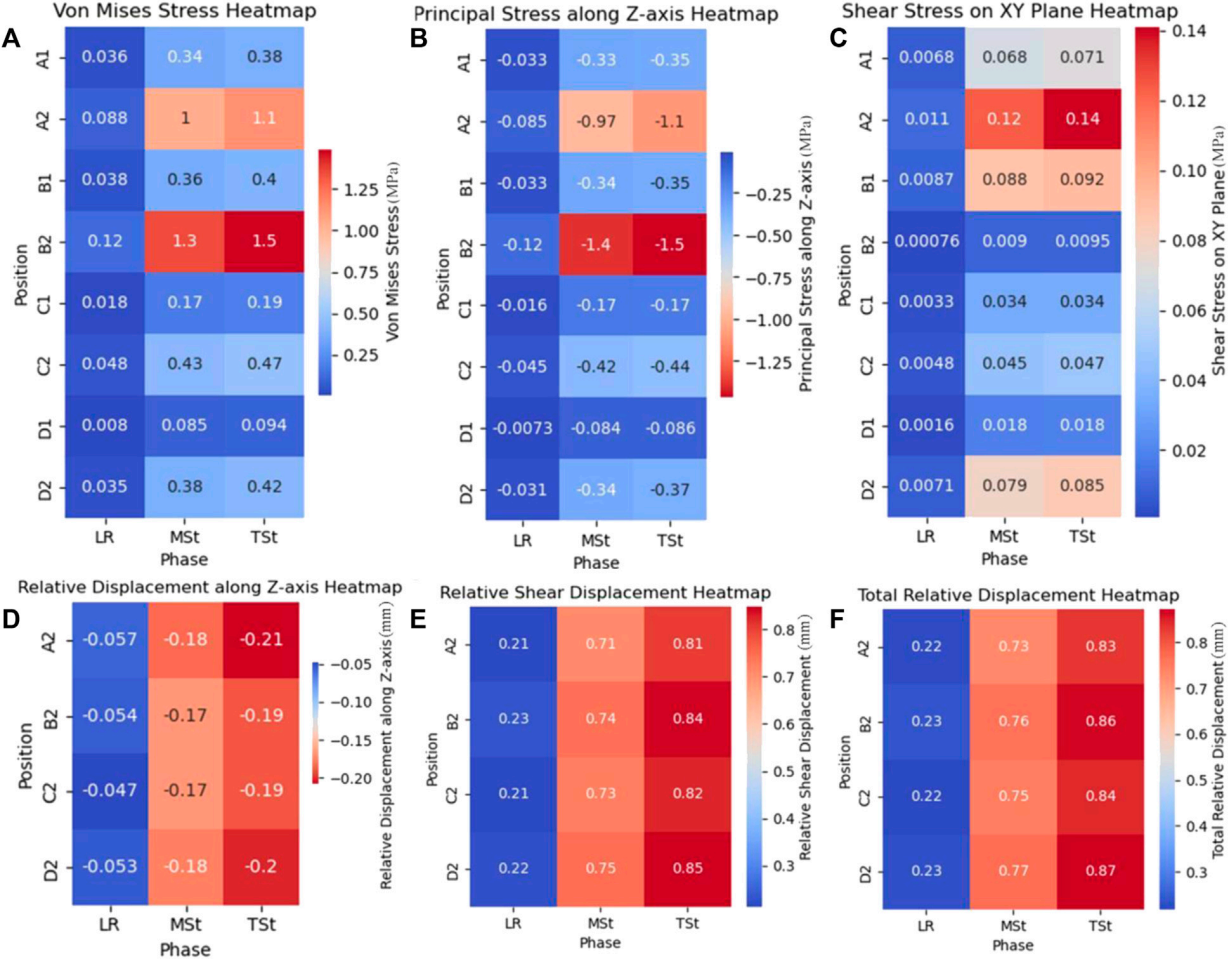
Stress and relative displacement heatmaps in first metatarsal osteotomy during three phases.**(A)**: Von Mises stress on osteotomy surfaces; **(B)** Principal stress along *Z*-axis on osteotomy surface; **(C)** Shear Stress on XY plane of osteotomy surfaces; **(D)** Relative displacement along *Z*-axis on distal osteotomy surface; **(E)** Relative shear displacement, also called relative displacement on XY plane, on distal osteotomy surface; **(F)** Total relative displacement (the vector sum of the relative displacements along three axes.) on distal osteotomy surface. Abbreviation Explanations: LR - Load response, MSt- Mid Stance phase. TSt- Terminal Stance.

Table 2; Figures 4, 5 presents the computed stress results at eight seleted nodes, including the axial stresses along the X, Y, and *Z*-axes, the shear stresses across three orthogonal planes, and the Von Mises stresses, each simulated during the LR, MSt, and TSt phases.

**TABLE 2:** Von mises, principal, and shear stresses at the osteotomy surface nodes (MPa).

Phase	Position	Von mises stress	Principal stress			Shear stress		
			*X*-axis	*Y*-axis	*Z*-axis	XY plane	Yz plane	XZ plane
LR	A1	0.036	−0.00515	0.004676	−0.03314	0.006814	0.000232	0.000697
	A2	0.088	−0.00857	0.008147	−0.08486	0.010882	0.001616	0.001215
	B1	0.038	−0.00507	0.005289	−0.03347	0.008713	0.00114	0.001143
	B2	0.118	−0.00428	0.006217	−0.11662	0.000755	0.001541	0.000773
	C1	0.018	−0.0027	0.003148	−0.01604	0.003277	0.000223	0.000671
	C2	0.048	−0.00621	0.007443	−0.04507	0.00482	0.000738	0.001234
	D1	0.008	−0.00101	0.00101	−0.0073	0.00157	0.0002	0.000201
	D2	0.035	−.00523	0.00585	−0.031	0.007104	0.000207	0.000416
MSt	A1	0.341	−0.05106	0.046333	−0.32835	0.067518	0.002297	0.006912
	A2	1.035	−0.09761	0.092787	−0.96648	0.123944	0.018404	0.013841
	B1	0.363	−0.05116	0.053384	−0.33781	0.08794	0.011509	0.011541
	B2	1.349	−0.05114	0.074246	−1.39266	0.009016	0.018408	0.00923
	C1	0.171	−0.02793	0.032528	−0.16576	0.033858	0.002304	0.006932
	C2	0.429	−0.05811	0.0696	−0.42152	0.045077	0.006902	0.011536
	D1	0.085	−0.01157	0.011549	−0.0835	0.017952	0.002291	0.002297
	D2	0.380	−0.05812	0.064976	−0.34436	0.078903	0.002301	0.004616
TSt	A1	0.376	−0.05382	0.048836	−0.34609	0.071165	0.002422	0.007285
	A2	1.140	−0.11102	0.105535	−1.09926	0.140972	0.020932	0.015743
	B1	0.400	−0.05335	0.055675	−0.3523	0.091713	0.012003	0.012036
	B2	1.485	−0.05389	0.078242	−1.46762	0.009501	0.019399	0.009726
	C1	0.188	−0.02823	0.032878	−0.16755	0.034222	0.002329	0.007006
	C2	0.472	−0.0611	0.073187	−0.44324	0.0474	0.007258	0.01213
	D1	0.094	−0.01189	0.011865	−0.08578	0.018443	0.002353	0.00236
	D2	0.418	−0.06249	0.069866	−0.37027	0.084842	0.002475	0.004963

Abbreviation Explanations: LR, Load response, MSt- Mid Stance phase. TSt- Terminal Stance.

The analysis of Von Mises stress during the three gait phases revealed that the maximum Von Mises stress consistently occurred at node B2, with values of 0.118 MPa for LR, 1.349 MPa for MSt, and 1.485 MPa for TSt. For the principal stress, the maximum values during the three phases were all compressive stresses along the *Z*-axis of node B2, increasing with changes in gait, and were recorded as −0.11662 MPa for LR, −1.39266 MPa for MSt, and −1.46762 MPa for TSt. Lastly, the analysis of shear stress indicated that the maximum shear stresses during the three phases were all located at node A2 on the XY plane, with values of 0.010882 MPa for LR, 0.123944 MPa for MSt, and 0.140972 MPa for TSt.

### 3.3 Displacement outcomes from FEA

Utilizing the axial displacements of nodes at various stages as derived from the FEA (Figures 4, 5), we computed the relative axial displacements and the relative total displacements of the nodes on the distal osteotomy surface, such as the displacement of node A1 relative to node A2 (Table 3). The results indicate that all axial relative displacements are negative, with the maximum relative displacements for the four pairs of nodes occurring during TSt. The respective relative total displacements for these pairs are 0.832, 0.865, 0.845, and 0.872.

**TABLE 3:** Relative displacements on the osteotomy surface nodes (mm).

Phase	Position	*X*-axis	*Y*-axis	*Z*-axis	XY plane	Total
LR	A2	−0.152	−0.152	−0.057	0.215	0.222
	B2	−0.175	−0.146	−0.054	0.228	0.234
	C2	−0.162	−0.138	−0.047	0.213	0.218
	D2	−0.167	−0.148	−0.053	0.223	0.229
MSt	A2	−0.503	−0.502	−0.184	0.711	0.734
	B2	−0.572	−0.476	−0.168	0.744	0.763
	C2	−0.554	−0.472	−0.168	0.728	0.747
	D2	−0.559	−0.496	−0.177	0.747	0.768
TSt	A2	−0.570	−0.569	−0.208	0.805	0.832
	B2	−0.649	−0.540	−0.190	0.844	0.865
	C2	−0.627	−0.534	−0.190	0.824	0.845
	D2	−0.634	−0.563	−0.202	0.848	0.872

Abbreviation Explanations: LR, Load response, MSt- Mid Stance phase. TSt- Terminal Stance.

## 4 Discussion

Increased acceptance of minimally invasive techniques for hallux valgus treatment is accompanied by ongoing debates over fixation methods, particularly external bandaging. Long-term studies (Sun et al., 2010; Wen et al., 2021) have demonstrated the clinical benefits of “8” bandaging. However, biomechanical evidence remains sparse, with limited and inconsistent finite element studies on postoperative fixation. Existing research using static FEA isn’t able to model the dynamic weight-bearing walking after surgery (Mao et al., 2017; Xie et al., 2023).

Our study integrates gait analysis with quasi-static FEA to simulate the biomechanics of weight-bearing walking post-surgery, providing biomechanical support for the treatment’s efficacy. The quasi-static FEA models and selects representative load points derived from gait analysis, defining respective loads to simulate various states, followed by static finite element analyses for each condition. This method, while avoiding the computational intensity of dynamic FEA, effectively captures the trends and ranges observed in dynamic models. Thus, quasi-static FEA shows significant promise for elucidating the biomechanical characteristics after orthopedic surgeries, offering a valuable tool for understanding postoperative dynamics without the computational burden of fully dynamic simulations.

In this study, the von Mises stress was employed to evaluate the risk of fixation failure or fracture under complex stress conditions. The maximum von Mises stress observed, occurring at time TSt and primarily distributed on the dorsal side, reached a peak value of 1.485 MPa. This value is lower than the yield strength of cancellous bone and also lower than the von Mises stress (7.8615 MPa) associated with bandage fixation failure reported by Xie et al. Consequently, the risk of fracture or fixation failure under the “8” shaped bandage configuration is extremely low.

Principal stress was utilized to assess the magnitude and direction of stress at the osteotomy site, further analyzing its impact on fracture healing. In this study, the principal stress at all nodes was predominantly compressive along the *Z*-axis. The maximum principal stress was recorded at node B2 during TSt (1.46762 MPa), and the minimum at node D1 during LR (0.0073 MPa). Appropriate axial compressive stress aids in maintaining the stability of the osteotomy site and promotes fracture healing.

The role of shear stress in bone healing remains controversial. Previous studies generally suggest that the presence of shear stress impedes bone healing (Ullah et al., 2020). However, other studies have proposed that appropriate shear stress can promote the rapid ossification of the external callus (Wittkowske et al., 2016). In this study, the maximum shear stress at all three time points was observed at node A2 on the XY plane, with the highest shear stress occurring at TSt (0.140972 MPa). Nevertheless, due to the lack of clear thresholds for the impact of shear stress on bone healing in existing research, the significance of shear stress will be discussed in conjunction with displacement in the following sections.

The results indicate that the “8” bandage effectively maintains the dynamic stability of the osteotomy site during the gait cycle, particularly during the stance phase where the majority of weight-bearing occurs. The consistent compressive stresses along the *Z*-axis and the lateral displacement of the osteotomy’s distal end suggest that the bandage provides adequate support to prevent excessive movement and maintain alignment, which is essential for proper healing.

In this FEA, constraints on the ankle joint surface resulted in displacement or rotation at both the proximal and distal ends of the first metatarsal under load. Thus, analyzing the relative displacement between the distal osteotomy site and the proximal osteotomy site is more significant. Nodes on the distal site (A1, B1, C1, D1) correspond to nodes on the proximal site (A2, B2, C2, D2). Relative displacement is represented by the absolute displacement difference between each pair of nodes, and the total relative displacement is calculated accordingly. Axial compressive displacement is widely acknowledged to promote bone healing (Hente et al., 2004). In this study, the relative *X*-axis displacement was consistently negative, indicating posterior movement along the metatarsal axis, which can promote callus formation and enhance stability at the osteotomy site.

Shear displacement (XY plane displacement) and total displacement are also crucial. The role of shear displacement in bone healing is debated; some researchers claim it hinders healing (Augat et al., 2003), while others find that limited shear displacement can enhance stability (Epari et al., 2007). Schell et al. (2005) found that with a 3 mm gap and axial displacement less than 0.5 mm, shear displacement below 8 mm aids bone healing. In this study, shear displacement increased during gait phases, peaking at 0.848 mm at node D2 during the terminal stance (TSt) phase, close to Schell’s proposed threshold.

The impact of total relative displacement on bone healing is also contested. Hu et al. (2022) suggested that relative displacements between 0.15 mm and 1.00 mm within a 3 mm gap benefit fracture healing. In this study, the minimum relative total displacement was 0.218 mm during the loading response (LR) phase, and the maximum was 0.871 mm during the TSt phase, both within Hu’s proposed range. Thus, based on FEA displacement results, the “8” shaped bandage external fixation maintains osteotomy site stability and effectively promotes bone healing.

Previous studies have compared the stability provided by “8” bandage fixation with that of internal fixation methods such as screws, plates, and K-wires. While some static FEA studies suggest that external bandage fixation may offer less effective stabilization compared to internal methods, our dynamic analysis under weight-bearing conditions demonstrates that the “8” bandage can indeed provide sufficient stability. This is particularly important as external fixation methods like the “8” bandage avoid complications associated with metal implants, such as skin irritation and the need for subsequent removal surgeries.

The results of this study support the use of “8” bandage fixation in minimally invasive hallux valgus surgery, especially considering its advantages in patient comfort, cost-effectiveness, and immediate weight-bearing capabilities. Clinicians should consider these findings when deciding on the most appropriate fixation method for their patients, particularly those who may benefit from avoiding metal implants or who prefer a more cost-effective solution.

## 5 Limitations and future research

While the current study offers valuable insights into the post-operative stability of the osteotomy site following hallux valgus correction with “8” shaped bandage fixation, it is subject to several limitations. First, the analysis is based on a single subject, which limits the generalizability of the findings. Future research should include a larger, more diverse sample with varying ages, genders, and body mass indices to enhance the applicability of the results.

Second, the lack of comparative analysis with other fixation methods restricts a comprehensive understanding of the relative efficacy of the “8” bandage. Third, the quasi-static FEA used in this study simulates conditions under slow loading but fails to account for dynamic aspects such as inertial effects and time-dependent behavior during gait. A dynamic FEA could provide a more accurate representation of real-world biomechanics.

Regarding the selection of load points, it is important to consider another important foot support pattern, which is the toe tips pattern that occurs after the forefoot support pattern. The stress at the three selected load points does not show a decline by the time of TSt, suggesting that the most challenging moment for the stability of the osteotomy site may not have occurred yet and could potentially arise during the toe-off phase. However, the toe tips pattern shows high variability. The structures ultimately lifted off the ground might include the big toe and the first metatarsal head, solely the big toe, or the big toe in conjunction with the second toe, among other configurations. This variability complicates the identification of characteristic points in this contact pattern. Future comprehensive dynamic biomechanical studies may reveal these dynamics.

Additionally, the constraint applied to the ankle joint surface in the finite element model, which fully restricts the ankle’s motion, may limit the accuracy of the simulations by not accounting for the natural variability in ankle joint angles during gait.

Moreover, the use of generic body weight values for simulations at the three load points may not precisely represent individual biomechanical conditions. Finally, the simulation of the “8” bandage’s fixation effect based on skin pressure measurements may not fully capture the external fixation dynamics. A more detailed approach to modeling the bandage’s influence is necessary.

## 6 Conclusion

This study has successfully established a finite element model of the “8” bandage fixation, integrating traditional Chinese and western medicine for hallux valgus treatment. By employing quasi-static FEA, the model effectively simulated and analyzed the biomechanical properties of the external fixation during the gait cycle. The findings suggest that the “8” bandage fixation system is a viable and effective method for stabilizing the osteotomy site following minimally invasive hallux valgus surgery. The dynamic stability provided by the bandage during the gait cycle supports its use in clinical practice, offering a less invasive and more cost-effective alternative to traditional metal fixation methods.

## Data Availability

The original contributions presented in the study are included in the article/Supplementary Material, further inquiries can be directed to the corresponding authors.
